# Robotic Assessment of Wrist Proprioception During Kinaesthetic Perturbations: A Neuroergonomic Approach

**DOI:** 10.3389/fnbot.2021.640551

**Published:** 2021-03-01

**Authors:** Erika D'Antonio, Elisa Galofaro, Jacopo Zenzeri, Fabrizio Patané, Jürgen Konczak, Maura Casadio, Lorenzo Masia

**Affiliations:** ^1^Assistive Robotics and Interactive Exosuits (ARIES) Laboratory, Institute of Computer Engineering (ZITI), University of Heidelberg, Heidelberg, Germany; ^2^Department of Informatics, Bioengineering, Robotics, and System Engineering (DIBRIS), University of Genoa, Genoa, Italy; ^3^Robotics, Brain, and Cognitive Sciences Unit, Italian Institute of Technology, Genoa, Italy; ^4^Mechanical Measurements and Microelectronics (M3Lab) Lab, Engineering Department, University Niccolò Cusano, Rome, Italy; ^5^Human Sensorimotor Control Laboratory, University of Minnesota, Minneapolis, MN, United States; ^6^Faculty of Engineering, The Maersk Mc-Kinney Moller Institute, University of Southern Denmark (SDU), Odense, Denmark

**Keywords:** proprioception, robotic assessment, multi-joint, static perturbation, motor control, biomechanics

## Abstract

Position sense refers to an aspect of proprioception crucial for motor control and learning. The onset of neurological diseases can damage such sensory afference, with consequent motor disorders dramatically reducing the associated recovery process. In regular clinical practice, assessment of proprioceptive deficits is run by means of clinical scales which do not provide quantitative measurements. However, existing robotic solutions usually do not involve multi-joint movements but are mostly applied to a single proximal or distal joint. The present work provides a testing paradigm for assessing proprioception during coordinated multi-joint distal movements and in presence of kinaesthetic perturbations: we evaluated healthy subjects' ability to match proprioceptive targets along two of the three wrist's degrees of freedom, flexion/extension and abduction/adduction. By introducing rotations along the pronation/supination axis not involved in the matching task, we tested two experimental conditions, which differed in terms of the temporal imposition of the external perturbation: in the first one, the disturbance was provided after the presentation of the proprioceptive target, while in the second one, the rotation of the pronation/ supination axis was imposed during the proprioceptive target presentation. We investigated if (i) the amplitude of the perturbation along the pronation/supination would lead to proprioceptive miscalibration; (ii) the encoding of proprioceptive target, would be influenced by the presentation sequence between the target itself and the rotational disturbance. Eighteen participants were tested by means of a haptic neuroergonomic wrist device: our findings provided evidence that the order of disturbance presentation does not alter proprioceptive acuity. Yet, a further effect has been noticed: proprioception is highly anisotropic and dependent on perturbation amplitude. Unexpectedly, the configuration of the forearm highly influences sensory feedbacks, and significantly alters subjects' performance in matching the proprioceptive targets, defining portions of the wrist workspace where kinaesthetic and proprioceptive acuity are more sensitive. This finding may suggest solutions and applications in multiple fields: from general haptics where, knowing how wrist configuration influences proprioception, might suggest new neuroergonomic solutions in device design, to clinical evaluation after neurological damage, where accurately assessing proprioceptive deficits can dramatically complement regular therapy for a better prediction of the recovery path.

## Introduction

The term “proprioception,” introduced in the early twentieth century, refers to the self-perception of position, motion and orientation of the body or body segments (Goldscheider, 1898; Sherrington, 1907; Evarts, 1981). Proprioceptive signals arise from mechanoreceptors embedded in our joints, muscles, and tendons such as muscle spindles or Golgi tendon organs (Proske and Gandevia, 2012). In general, two submodalities of proprioception are distinguished: (i) *kinaesthesia*, the sense of limb movement; (ii) *joint position sense*, the sense of limb position (Proske, 2006). These two senses constitute the sensory stream colloquially referred to as conscious proprioception.

Neurological pathologies, such as stroke (Carey, 1995) or Parkinson's disease (Konczak et al., 2012), can permanently deprive the brain of its main sources of dynamogenic information from skin and muscles (Debert et al., 2012), leading to a compromised coding of the proprioceptive information, with negative consequences in motor control and the associated recovery progress (Marchal-Crespo and Reinkensmeyer, 2009; Schabrun and Hillier, 2009). Accurate assessment and quantification of proprioceptive function becomes a leading factor in the diagnosis and treatment of neurological diseases.

Despite the paramount importance of proprioceptive feedback in motor coordination and recovery (Raspopovic et al., 2014), actually, there are no established methods capable of assessing multi-joint proprioceptive acuity in a reliable, objective fashion. Recent advancements in robotic and haptic technology (Yeong et al., 2009; Oblak et al., 2010) represent the starting point for the development of automated, repeatable robot-aided methodology for studying proprioception and potentially provide standardized, quantitative methodology to evaluate kinaesthetic and proprioceptive performance characterized by a continuous ratio scale (Simo et al., 2014; Deblock-Bellamy et al., 2018; Klein et al., 2018; Mochizuki et al., 2019). In addition, the use of robotic devices to study sensory motor control should be designed considering anthropometric and biomechanical features, not only for what concerns the mechanical design but also for the implementation of the related control strategies (Chiri et al., 2012). These complementary characteristics (design & control) are paramount to exploit the real potential of robotic technology in both neuroergonomics, addressing general motor behavioral aspects, and clinical environment where robustness and reliability of such devices can be only reached starting their conception from human factors.

Although it has been demonstrated that proprioception of distal joints is particularly involved in fine manipulation of daily living activities (Hoseini et al., 2015; Ponassi et al., 2018), scientific literature primarily reports contributions focused on proprioception at the level of proximal upper limb (shoulder and elbow). Previous research focused on distal joints, with particular emphasis on wrist's proprioceptive functions (Aman et al., 2015; Rose et al., 2018). In particular, concerning our group, we extensively tested proprioceptive acuity using a device named WristBot (Masia et al., 2009), which allows for the implementation of a widely used test for the assessment of position sense (Cappello et al., 2015), the *Joint Position Matching* (JPM) paradigm (Goble, 2010): the test is run in absence of visual feedback and evaluates the proprioception by quantifying the accuracy in replicating a joint posture (proprioceptive target), previously imposed as angular displacement. Previous works investigated the wrist proprioception along a single degree of freedom (DoF) evaluating (Marini et al., 2016a) its anisotropy across wrist abduction/adduction (AA) and flexion/extension (FE) DoFs, as well as a gradual change of proprioceptive acuity during the developmental phase for individuals (Marini et al., 2017). However, proprioception for distal multi-joint movements, involving more than a single DoF, still remains an open question, and there is limited evidence in literature on the mechanism underlying the integration of proprioceptive sensory stream from multiple concurring anatomical joints (Sketch et al., 2018).

In daily manipulation tasks, the use of the wrist and hand requires a complex motion strategy between the fingers and the two distal DoFs corresponding to wrist FE and AA. Moreover, the forearm can rotate along its longitudinal axis by engaging a third wrist DoF, the pronation/supination (PS), which allows the hand to cover a wider workspace and exploit the arm's kinematic redundancy. The wrist biomechanics, almost unique among all human anatomical districts, allows an extremely efficient manipulation dexterity, as highlighted by the study of Kane et al. (2014), which showed how the combination of FE and AA ROMs results in a workspace which is independent from the rotations around the PS axis, being its motion completely disconnected from the previous wrist joints. Within the framework of the current study, we hypothesize that providing perturbations along the PS axis, consisting in rotational offset of variable amplitude along the forearm, will not lead to physical limitations on the remaining wrist DoFs and sensory conflicts in terms of proprioception acuity during joint position matching tasks. The multi-joint biomechanics of the wrist joint are known, yet the processing of proprioceptive information across its DoFs is less well understood. Proprioceptive efferent signals are encoded in reference frames localized at the level of joints (Flanders and Soechting, 1995): in order to compute motor commands, the central nervous system must process such sensory information and project it into a spatial representation of motion (Colby, 1998). Yet, movement generation relies on information redundancy by merging both visual and proprioceptive feedback, continuously streamed during a general task execution, and consequently integrating both absolute spatial and local sensory streams, respectively (Snyder et al., 1998). What happens if visual information is excluded from the integrative process and motion computation must rely on one sensory feedback? How, in such condition, an external disturbance, altering the encoding of proprioceptive information, influences the task performance? With this in mind, we designed an experiment to investigate if the sole proprioceptive information, can be robustly retained by the brain even in presence of a kinesthetic disturbance altering the geometric conditions between the presentation of the task and its execution.

How proprioceptive information is interpreted when complex wrist motions are performed, and whether multi-joint kinaesthetic sensory streams are encoded throughout the wrist workspace, are examples of unanswered questions crossing the domains of neurophysiology and clinical rehabilitation. Most studies involving multi-joint tasks, have primarily investigated distal arm goal directed movements toward visual targets: results suggest that the relative contributions of vision and proprioception to motor planning can change, depending on the modality in which task relevant information is represented (Sarlegna and Sainburg, 2007). Yet, all this extensive production of results has covered experimental paradigms deeply involving visual-feedback (Goble and Brown, 2009), while encoding of proprioceptive targets in coordinated tasks is still an open debate, especially for what concerns integration of proprioceptive information among the DoFs of a multi-joint articulation.

The goal of the present research is to investigate, using a neuroergonomic approach, the influence of wrist posture on proprioceptive acuity during multi-joint JPM tasks and under different perturbations. By imposing angular offset rotations in different fashions of amplitude and sequence on the DoF which is not involved in the matching task (PS), we tested proprioceptive acuity on the remaining wrist joints, with the purpose of providing insights on how (i) proprioception is encoded in a complex biomechanical structure, (ii) sensory information are integrated, and (iii) external disturbances are rejected.

## Methods

### Participants and Experimental Setup

Eighteen young healthy subjects (age 27.4 ± 2.8 years (Mean ± STD), 9 females) were recruited for the study: participants self-reported no evidence or known history of neurological disease and exhibited normal joints range of motion and muscular strength. To be included in the study subjects had to be right-handed, according to the Edinburgh Handedness Inventory (Oldfield, 1971) [EHI score > 60; EHI score = 81.89 ± 13.07 (Mean ± STD)]. The research was in accordance with the ethical principles of the 1964 Declaration of Helsinki, which protects research participants. Each subject signed a consent form conformed to these guidelines to participate in the study and to publish pseudonymized individual data. All the study procedures and documents were approved by the Heidelberg University Institutional Review Board (S-287/2020). Experiments were carried out at the Aries Lab (Assistive Robotics and Interactive Ergonomic Systems) of the Institute of Computer Engineering of Heidelberg University (Germany).

**Figure 1:**
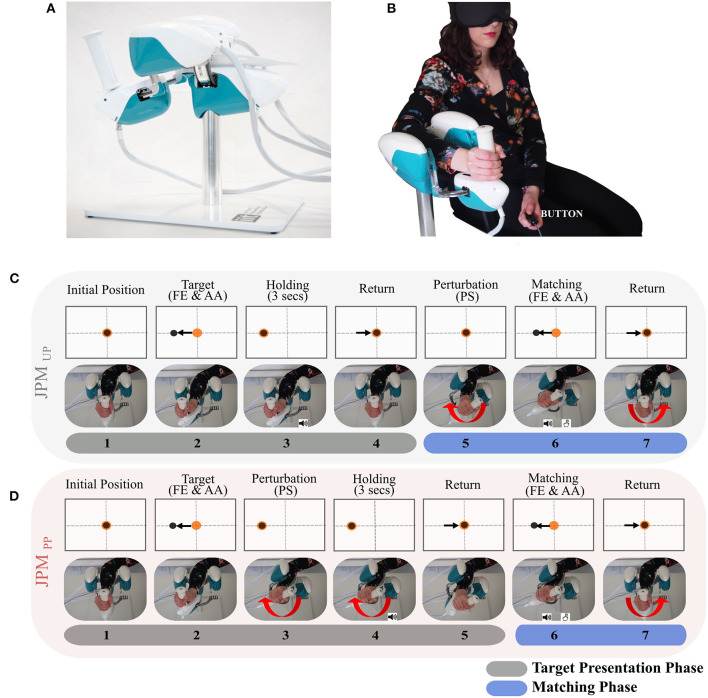
**(A)** The WristBot device. **(B)** Experimental setup. The subject is comfortably seated on a chair with the right forearm fixed on the WristBot robotic device while holding its handle. In the contralateral hand the subject holds the button to press during the proprioceptive “Matching Phase.” The subject wears a mask over his eyes to perform the experiment based only on his proprioceptive feedback. **(C,D)** are represented the temporal sequences for the two JPM conditions: JPMUPJPM_*UP*_ and JPM_*PP*_PM. From the initial position, the wrist joint is passively moved towards the proprioceptive target (passive reaching) and then maintained for 3 s. An auditory cue marks that the proprioceptive target is reached. After returning to the resting position participants are asked to match the target, as accurate as possible (Matching Phase) by pressing the button with the contralateral hand. Another auditory cue signals to the subject the start of the Passive Matching Phase in which it is required to stop the robot once the same movement amplitude has been perceived. In different temporal moments, depending on the condition experienced, a perturbation is given (angular rotation along the PS axis of a certain random amplitude). This is evidenced by the red arrow in the figure. Orange dot represents the device end-effector position, while the black dot represents the proprioceptive target position.

The experimental design involved a task, where subjects were sitting in front of a screen, holding the handle of a haptic device (WristBot) with their right hand (Figure 1A). Subjects were blindfolded during the whole experiment, but during a phase of familiarization the visual feedback was provided to explain the task sequence and how to perform it correctly.

The employed device has three DoFs: FE (± 62°); AA (+45/−40°); PS (± 60°) and it allows almost the full range of motion of the human wrist. It is driven by 4 brushless motors dimensioned in order to compensate for weight and inertia and to provide sufficient haptic rendering at the level of wrist. Angular rotations on the three axes are acquired by means of incremental encoders, resulting in a resolution of 0.17°. The continuous torque ranges at the different wrist joints are 1.57 Nm on FE, 3.81 Nm on AA, and 2.87 Nm on PS, Figure 1A. During the experiment, participants sat beside the robotic device with the frontal plane of their body aligned perpendicularly to the PS axis of the robotic device, Figure 1B. The position of each participant was carefully adjusted to ensure a 90° elbow angle and the correct alignment between the wrist and the robotic system axes, Figure 1B. Participants' trunk was not constrained, yet the forearm was secured in such a way that backrest ensures a 90° elbow angle, while hand position on the device's handle was kept constant over the course of the experiment and registered for each participant on her/his anthropometrics. Subjects forearm was strapped to a mechanical support using anatomical references (i) to ensure repeatability of wrist positioning, thus trying to limit inter-trial variability, (ii) to avoid joints misalignment, and (iii) involuntary relative movements between the device and the wrist during task execution. Moreover, the device's handle was carefully designed to be opportunely adaptable to the different subjects' anthropometrics, by means of a sliding system that allows to secure the forearm on the device.

### Task and Procedure

The protocol implemented explored how angular perturbations can affect sensory acuity and consequently altering proprioceptive thresholds. A similar experimental design has been described in Masia et al. (2009), where, in a point-to-point reaching task, rotational misalignments were applied between the visual (spatial) and the proprioceptive (local) frames, creating a visuo-proprioceptive miscalibration. We wanted to use a comparable paradigm applied to a single sensory feedback by using local rotations among the wrist degrees of freedom by changing the configurations between the presentation of the proprioceptive stimuli (target) and the matching task. In particular, we used the wrist rotation along the PS axis to provide the perturbation in the context of a passive JPM test, which was exploited using the remaining DoFs of the wrist (Goble, 2010; Marini et al., 2016b). The proprioceptive task consisted in an ipsilateral JPM along two DoFs of the wrist (FE and AA): from an initial rest position (0° of FE, 0° of AA and 0° of PS) a preset wrist stimulus or proprioceptive target, corresponding to about 50% of the total functional wrist ROM (Kim et al., 2014), was passively presented to a blindfolded participant, who was then asked to match it, as accurately as possible in a subsequent movement. In particular, these angles were: 32° for FE; 16° for AA (Marini et al., 2016a).

The perturbation delivered to participants during the JPM task consisted in seven pseudo randomized rotations along the PS axis (−45°, −20°, −5°, 0°, +5°, +20°, +45°), at speed equal to 12°/s and in two separate temporal fashions: depending on the time in which the perturbation was given, we distinguished two task conditions named *JPM*_*UP*_ (Unperceived Perturbation) and the *JPM*_*PP*_ (Perceived Perturbation), which will be explained in detail in the next paragraphs.

Each target set consisted of 48 repetitions (trials) for each of the two DoFs separately (FE and AA), for a total of 96 provided proprioceptive targets. It was divided into 2 sub-sets (20 min each), with a break of about 10 min, to avoid fatigue and loss of concentration.

Each single trial consisted in two separate phases indicated as “*Target Presentation Phase*” and “*Matching Phase*”: seven blocks composed the aforementioned phases and are depicted as a breakdown in Figures 1C,D (in the figure, only test on the FE is illustrated for sake of simplicity). From the initial wrist position (Block 1), the robot moved one DoF to the preset angular position corresponding to the proprioceptive target or stimulus (Block 2). An *auditory cue* (high-frequency beep) was provided when the robot reached the proprioceptive target: from this block onward, the trial can follow a different order of presentation depending on the two disturbance conditions, as explained as follows:

1. Condition *JPM*_*UP*_(Figure 1C): the current experimental condition is separated in three main events during each trial: *presentation of the proprioceptive target PS perturbation matching phase*.
  In details, each single trial in such condition started with the wrist of the participant in the physiological neutral configuration (Block 1), then the robot moved the wrist to a *proprioceptive target* (Block 2) along FE (or AA) and maintains such configuration for 3 s (Block 3) (Fuentes and Bastian, 2010). Successively, the subject's wrist is moved back to the initial *rest* configuration (Block 4); At this point a pseudo random *perturbation* around the PS axis (Block 5) was provided. An *auditory cue* indicated the initiation of the *Matching Phase*, where the rotated subject's wrist was passively moved by the robot toward the same direction of the previously presented target (Block 6) on FE (or AA). During this block subjects were instructed to stop the robot motion by pushing a *button* with the contralateral hand, as soon as they perceived to have reached a joint amplitude matching the one of the previously presented target. The robot speed was changed respect to the one experienced during the *proprioceptive target* presentation (Block 2), to prevent subjects from relying on the memory time factor during execution of the *matching phase*. At last, the robot drove back the subject's wrist to the initial position prior next trial initiation (Block 7).
2. Condition *JPM*_*PP*_ (Figure 1D): contrarily to the previous condition, we had 2 (and not 3) events: *presentation of the proprioceptive target including PS perturbation matching phase*.
  *The presentation of the target along FE (or AA) was passively imposed by the robot starting from a rest position (Block 1–2). At this point, contrarily to the previous condition, the pseudo random PS perturbation (Block 3) was presented while maintaining the target presentation on FE (or AA), held for 3 s (Block 4) and successively repositioning FE (or AA) to the rest configuration (Block 5): this was the end of the *Target Presentation Phase*. The *Matching Phase* started with the *passive matching* (Block 6): after an *auditory cue*, subjects were required to stop the robot motion, by pressing the button in the contralateral hand, once the same movement amplitude has been perceived. Immediately after pressing the button, the robot brought the subjects wrist back again to the initial position for the next trial (Block 7)*.

Subjects were instructed to focus only on the location of the proprioceptive target and try to reject the effect of the perturbation along the PS axis during the *Matching Phase*. They did not receive any feedback about their performance, to eliminate a possible recalibration of the responses during the test. Across 2 days of testing (day 1 and day 2), participants were required to perform the task in a randomized order for the two conditions *JPM*_*UP*_ (day 1 or day 2) and *JPM*_*PP*_ (day 1 or day 2).

### Data Analysis

Wrist joint rotations were recorded by means of the digital encoders of the WristBot (data collection frequency set at 100 Hz). Data were filtered offline using a 3rd order Savitzky–Golay low-pass filter (cut-off frequency of 10 Hz). For each condition, as a measure of the overall accuracy, we computed two indicators: the *error bias* and the *matching error* (Schmidt et al., 2018).

- The *error bias* ([°]), is the mean, over N repetitions for the same proprioceptive target (same DoF and disturbance condition), of the signed difference between the presented proprioceptive target location (ϑ_*target*_) and the wrist position at the end of the matching task movement (ϑ_*i*_). It indicates the subject's tendency to overshoot (positive *error bias*) or undershoot (negative *error bias*) the target after the *Matching Phase*. For a consistent interpretation, we transformed the signed *error bias* to a measure of a signed overshoot, *error bias*_*OS*_ (Galofaro et al., 2019):

(1) $$\begin{array}{r} error\;bias{\;}_{OS}=sign\left(\vartheta_{target}\right)\;*\;\frac{\sum_{i=1}^{N}(\vartheta_{i}-\vartheta_{target})}{N} \end{array}$$

where ϑ_i_ is the measured value at the end of the *i-th* trial, ϑ_target_ is the target position. In this metrics, negative values represent an undershoot, while positive values represent an overshoot independently of the sign of the target.

- The *matching error* ([°]), evaluates the accuracy during the *Matching Phase* and it is defined as the absolute value of the difference between the ϑ_i_ and the ϑ_target_ averaged over N repetitions of the same target in the same disturbance condition:

(2) $$\begin{array}{r} matching\;error=\frac{\sum_{i=1}^{N}{|\vartheta }_{i}-\vartheta_{target}|}{N} \end{array}$$

### Statistical Analysis

Data normality distribution was assessed using Shapiro-Wilk test, and sphericity condition for repeated measures analyses of variance (rANOVA) was assessed using the Mauchly test. The first test was always verified: when the second was violated, we applied the Greenhouse-Geisser correction. The three-way repeated measures ANOVA test was used to examine the effects, on the dependent variables (*error bias, matching error*) of the robot rotation around the PS axis, the DoF and the tasks condition, using three within-subject factors: (i) ‘condition' (2 levels: JPM_PP_ and JPM_UP_), (ii) ‘PS perturbation' (7 levels: −45°,−20°, −5°, 0°, 5°, 20°, 45°), (iii) 'DoF' (2 levels: AA and FE) and their interaction. A *post-hoc* analysis was performed using Paired *t*-tests to evaluate the significant pairwise differences between each perturbation, DoF and condition. For all the tests, the level of statistical significance was set at 0.05, except for *post-hoc* analysis, where the significance level was chosen according to the Bonferroni correction for multiple comparisons. Statistical analysis was conducted by using IBM SPSS Statistics 23 (IBM, Armonk, New York, USA).

## Results

### Comparison Between JPM_UP_ and JPM_PP_

**Figure 2:**
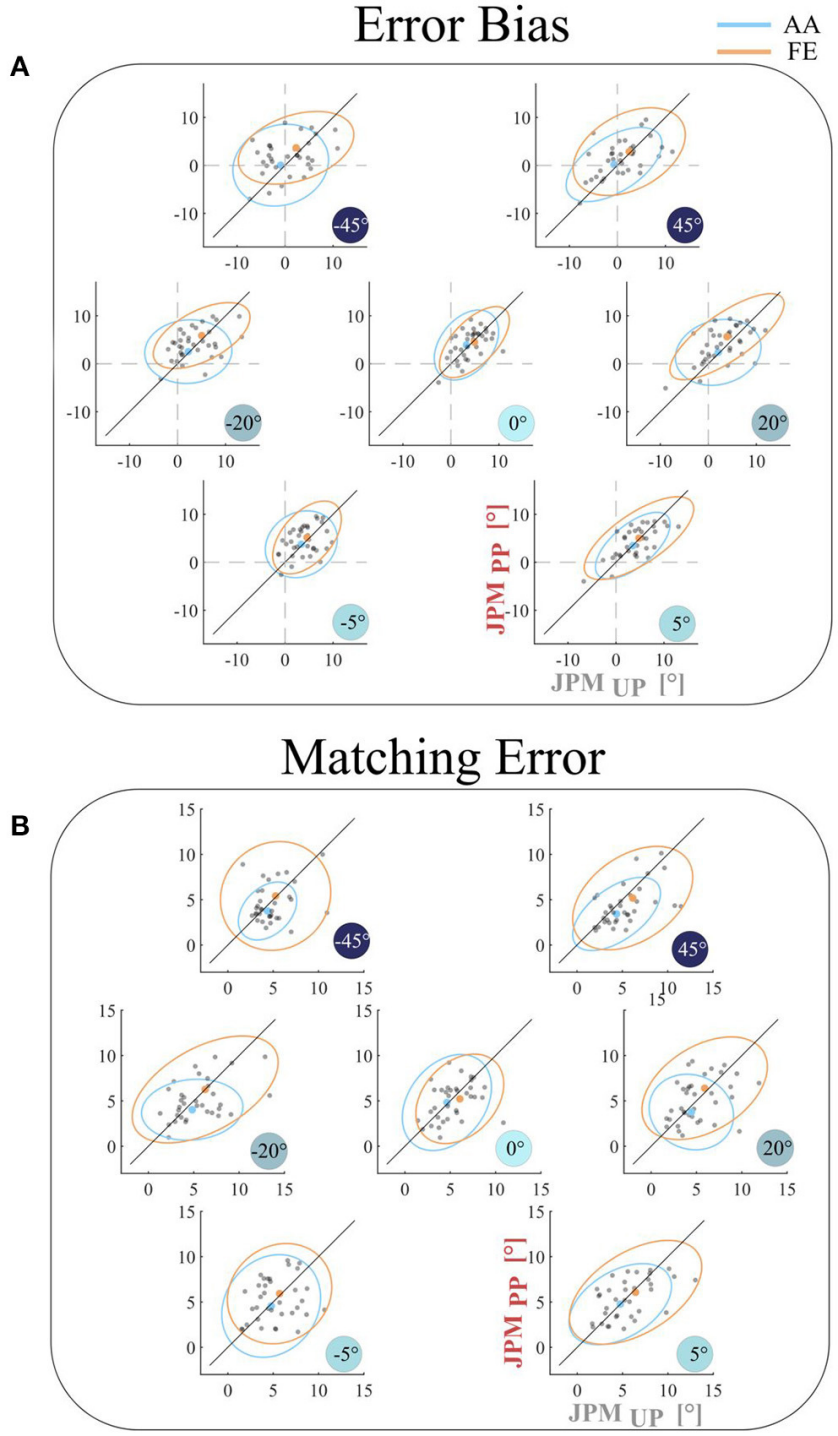
Comparison between the two experimental conditions (*JPM*_U*P*_ vs. *JPM*_P*P*_) for the **(A)** *Error Bias* and for the **(B)** *Matching Error* outcomes for AA (light blue) and FE (orange) DoFs. Each gray point represents the average result for a single subject. The mean result across the population is reported as light blue point for AA and orange point for FE joint. The line through the origin (equality line) is represented by a black line; if the subject performance stays above this line the error is higher for the *JPM*_P*P*_ task, vice versa if it stays under the line.

Figure 2 shows the comparison between the two disturbance conditions (*JPM*_*UP*_ vs. *JPM*_*PP*_) in terms of the *error bias* (A) and the *matching error* (B). As evidenced also by the rANOVA results, for both outcomes, we did not find any significant difference between the two conditions (*JPM*_*UP*_ vs. *JPM*_*PP*_; *error bias: F* = 0.986, *p* = 0.329; *matching error*: *F* = 1.424, *p* = 0.211 F). *Error bias* and *Matching Error* indicated that the performance, averaged across all subjects and independently on the investigated DoF (FE and AA), it's closely distributed along the *equality line*, demonstrating that the process underlying encoding of proprioceptive target is not influenced by the order of rotation of the reference frames between *target presentation* and *matching movement*. Moreover, the same behavior persists across all the spanned values of the PS perturbation.

### Effects of Pronation/Supination Disturbance on Over- and Under-Shooting the Proprioceptive Targets

**Figure 3:**
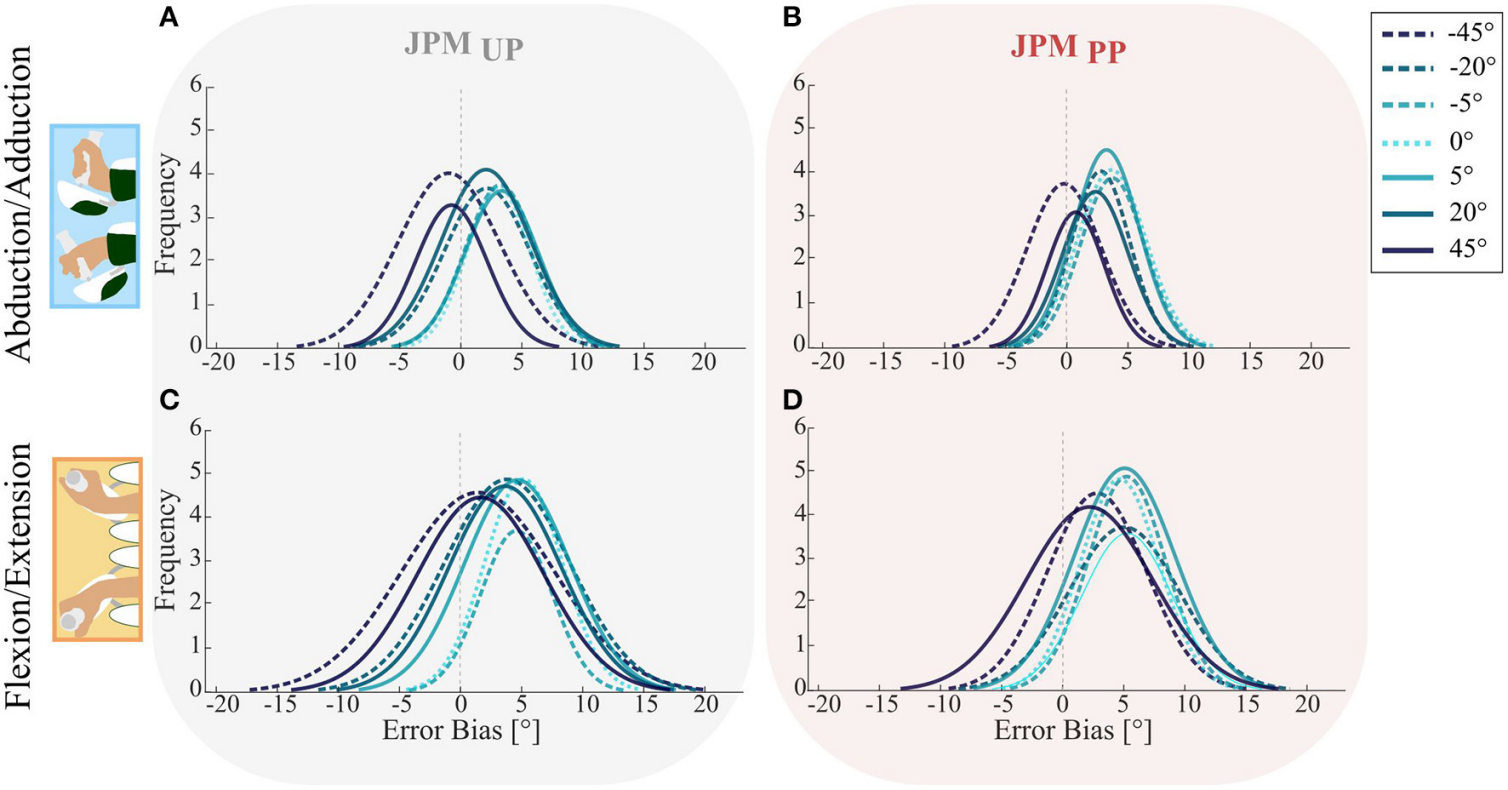
Probability density distributions for the Error Bias of the two DoFs AA **(A,B)** and FE **(C,D)** in both the *JPM*_U*P*_ (first column) and *JPM*_P*P*_ (second column) conditions. Colored lines show the mean distribution for the specific perturbation denoted in the legend. The vertical dotted line highlights the error equal to zero, a distribution shifted to the left indicates error undershooting, while a distribution shifted to the right represents a tendency of target overshooting.

The trend of the subjects to overshoot or undershoot the angular position of the proprioceptive target during the *Matching Phase* was examined by analyzing the probability density distribution of the *error bias* for across the two investigated DoFs FE and AA (Figure 3). We evaluated the distribution for the 7 amplitude pseudo-random perturbations along PS and for both the *JPM*_*UP*_ and the *JPM*_*PP*_ conditions. The tendency to overshoot the proprioceptive target during the matching task was higher for low amplitude PS perturbations, rather than for the largest ones (−45° and +45°) in both tested DoFs (FE and AA). As previously reported in section Comparison between *JPM*_*UP*_ and *JPM*_*PP*_, also in this metric the two conditions (*JPM*_*UP*_ and *JPM*_*PP*_) did not influence the *error bias*. Task execution along the AA axis (Figures 3A,B) shows a tall narrow distribution mainly shifted to the right side for the perturbations which are closer to the physiological neutral posture of the wrist (0, ∓5°, ∓20°). For large PS perturbations (∓45°), the distributions were mainly centered around zero *error bias*, indicating a better matching performance of the proprioceptive target. As for the FE task, the results were similar, although characterized by a less distinct, behavior: for both the target presentation conditions (Figures 3C,D) subjects tended to overshoot the proprioceptive targets, but with a more accurate matching for those perturbations at the boundaries of the workspace (∓45°), rather than in configurations (0, ∓5°, ∓20°) close to the neutral position of wrist.

The aforementioned differences related to *Error Bias* were confirmed by the rANOVA highlighting a significant effect of the PS perturbation (*F* = 22.939, *p* < 0.001), and DoF (*F* = 37.199, *p* < 0.001), but not their interaction effect ('PS perturbation^*^ DoF' effect *F* = 1.198, *p* = 0.312).

**Figure 4:**
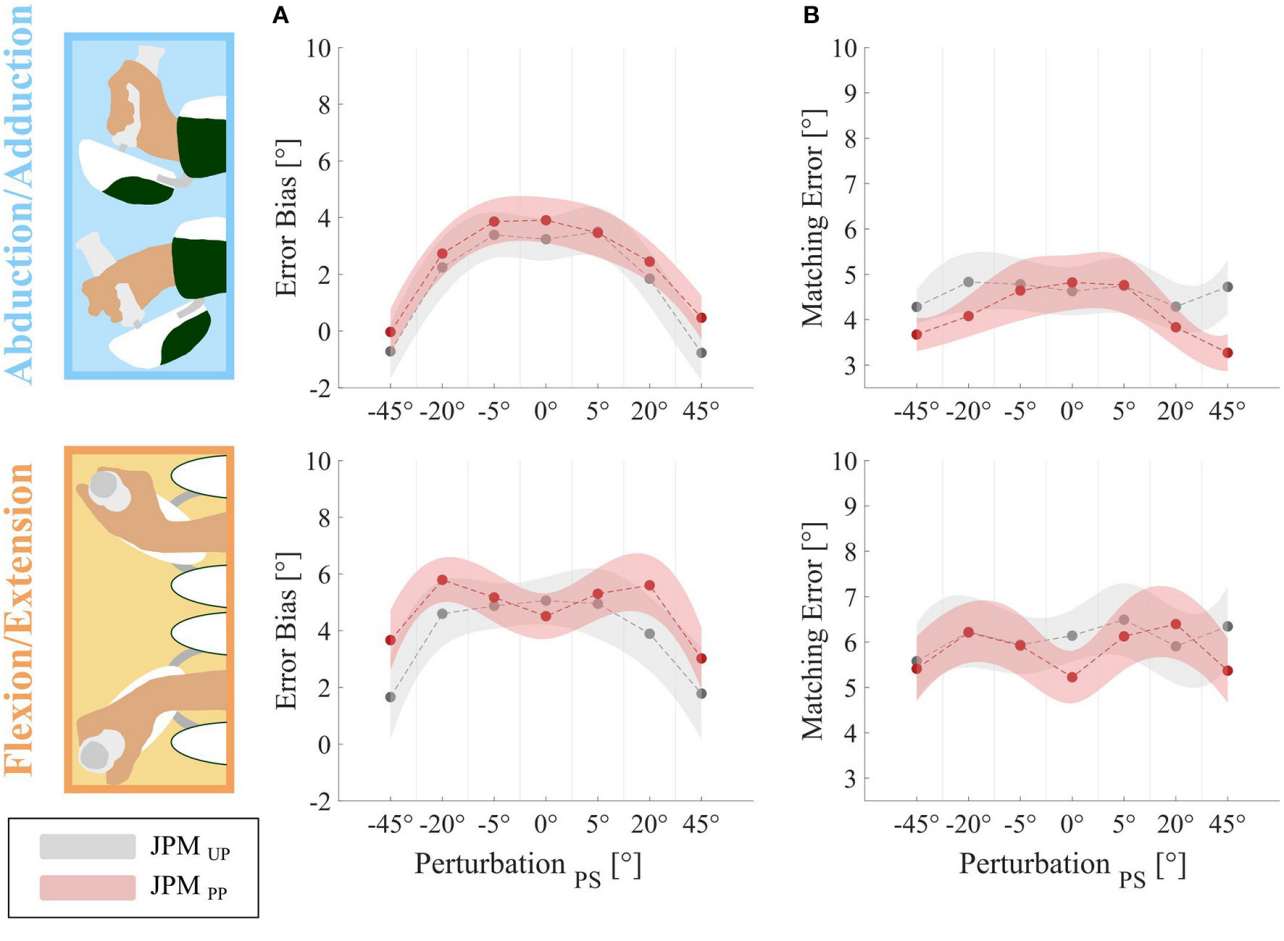
Outcome measures relative to the two DoFs: AA on the top and FE on the bottom for *JPM*_U*P*_ (gray) and *JPM*_P*P*_ (magenta) conditions. First column represents the *Error Bias* **(A)**. Second column is relative to the *Matching Error* **(B)**. On the x-axis is evidenced the amount of angular perturbation provided along the PS axis (−45°, −20°, −5°, 0°, 5°, 20°, 45°) during the experiment.

We statistically inferred the role of PS perturbation amplitude by a paired *t*-test *post-hoc* analysis for the *Error Bias*, and it revealed multiple significant differences (see Table 1). In particular, for all perturbations' amplitudes with the exception of the case related to the DoF FE and the condition *JPM*_*PP*_, we found an overshoot inversely proportional to the PS amplitude as visible by a bell shape graph (Figure 4A).

**Table 1:** Statistical *p*-values for the error bias between the seven perturbations.

		*******JPM*******_*********UP*********_		*******JPM*******_*********PP*********_	
**PS [**^****°****^**]**		**P (AA)**	**P (FE)**	**P (AA)**	**P (FE)**
**45**					
	20	<0.001*	0.007*	<0.001*	<0.001*
	5	<0.001*	<0.001*	<0.001*	0.003*
	0	<0.001*	<0.001*	<0.001*	0.008*
	−45	0.558	0.877	0.389	0.532
	−20	<0.001*	0.012*	0.002*	0.002*
	−5	<0.001*	0.001*	<0.001*	0.001*
**20**					
	5	0.007*	0.026*	0.015*	0.636
	0	0.011*	0.015*	<0.001*	0.200
	−45	<0.001*	0.013*	<0.001*	0.001*
	−20	0.721	0.614	0.884	0.355
	−5	0.011*	0.169	0.001*	0.838
**5**					
	0	0.569	0.430	0.245	0.171
	−45	<0.001*	<0.001*	<0.001*	0.01*
	−20	0.043*	0.284	0.186	0.734
	−5	0.796	0.909	0.213	0.855
**0**					
	−45	<0.001*	<0.001*	<0.001*	0.017*
	−20	0.05	0.155	0.020*	0.270
	−5	0.705	0.426	0.863	0.136
**−45**					
	−20	<0.001*	<0.001*	0.001*	0.002*
	−5	<0.001*	0.001*	<0.001*	0.006*
**−20**					
	−5	0.016*	0.620	0.005*	0.891

* *represents significant differences between the two perturbations compared*.

At last, a *post-hoc* analysis between the two tested DoFs, is reported in Table 2 for the *Error Bias* outcome: we found a significant difference between FE and AA for all the perturbations except for the condition *JPM*_*PP*_ at 0° of PS. In particular, subjects presented a larger overshoot along the FE DoF, for all the perturbations and conditions.

**Table 2:** Statistical *p*-values for the error bias between the two DoFs (AA/FE).

	**UP**		**PP**	
**PS [^**°**^]**	**Mean ± SD [^**°**^]**	***p***	**Mean ± SD [^**°**^]**	***p***
**−45**	**−0.72** **±** **4.09**	0.016*	**0.39** **±** **3.06**	<0.001*
	1.66 ± 6.21		3.76 ± 4.55	
**−20**	**2.24** **±** **4.40**	0.002*	**3.31** **±** **3.06**	0.002*
	4.59 ± 4.92		5.84 ± 3.19	
**−5**	**3.39** **±** **3.42**	0.004*	**3.51** **±** **3.18**	0.035*
	4.88 ± 3.45		5.05 ± 3.65	
**0**	**3.24** **±** **3.22**	<0.001*	**3.65** **±** **3.32**	0.197
	5.06 ± 3.54		4.51 ± 3.42	
**5**	**3.50** **±** **3.67**	0.034*	**3.26** **±** **3.55**	0.010*
	4.95 ± 5.23		5.30 ± 3.85	
**20**	**1.84** **±** **4.05**	0.013*	**2.36** **±** **3.17**	<0.001*
	3.89 ± 5.56		5.33 ± 4.28	
**45**	**−0.77** **±** **4.10**	0.018*	**0.76** **±** **3.03**	0.007*
	1.78 ± 6.90		3.02 ± 4.74	

* *represents significant differences between the two compared DoFs. Bold values represent AA axis*.

### Proprioceptive Anisotropy Related to the Perturbation Amplitude

In order to explore the distribution of proprioceptive acuity over the different PS perturbation amplitudes and across the two DoFs, we analyzed the *Matching Error* trend (Figure 4B).

The rANOVA showed on *Matching Error* showed a significant main effect of the DoF (FE vs. AA) (*F* = 44.695, *p* < 0.001) as well as of the PS perturbation amplitude (*F* = 3.025, *p* = 0.008). Detailed numerical outcomes of the *post-hoc* analysis across the two DoFs are reported in Table 3: again on the *Matching Error*, a significant difference between FE and AA was found for almost all the perturbations with the exception of 0° for the *JPM*_*PP*_ and −45° for the *JPM*_*UP*_. In particular, for both the conditions *JPM*_*UP*_ and *JPM*_*PP*_ and for all the PS amplitudes, subjects showed a larger *Matching Error* along the FE than the AA (Table 3), indicating an anisotropy of proprioceptive acuity across two DoFs which persists independently on the provided perturbations.

**Table 3:** Statistical *p*-values for the matching error between the two DoFs (AA/FE).

	**UP**		**PP**	
**PS [^**°**^]**	**Mean ± SD [^**°**^]**	***p***	**Mean ± SD [^**°**^]**	***p***
**−45**	**4.81** **±** **2.65**	0.053	**3.67** **±** **1.55**	0.001*
	5.95 ± 4.00		5.68 ± 3.10	
**−20**	**4.83** **±** **2.72**	0.012*	**4.18** **±** **1.97**	<0.001*
	6.45 ± 3.55		6.73 ± 3.05	
**−5**	**4.78** **±** **2.42**	0.014*	**4.64** **±** **2.79**	0.008*
	5.94 ± 2.73		6.14 ± 3.11	
**0**	**4.63** **±** **2.80**	<0.001*	**4.82** **±** **2.56**	0.128
	6.50 ± 3.54		5.59 ± 2.83	
**5**	**4.75** **±** **2.59**	0.005*	**4.76** **±** **2.63**	0.002*
	6.49 ± 3.44		6.62 ± 3.47	
**20**	**4.53** **±** **2.64**	0.022*	**3.75** **±** **1.86**	<0.001*
	5.91 ± 3.52		6.15 ± 3.03	
**45**	**4.13** **±** **2.04**	0.005*	**3.36** **±** **1.74**	<0.001*
	6.22 ± 3.73		5.84 ± 3.31	

* *represents significant differences between the two compared DoFs. Bold values represent AA axis*.

The *post-hoc* analysis between PS amplitudes for the *Matching Error* are reported in Table 4 and highlighted significant differences for the *JPM*_*PP*_ and the AA DoF. For all perturbations' amplitudes, we found a proprioceptive error inversely proportional to the PS amplitude as visible by a bell shape graph, Figure 4B. For large PS perturbations (∓45°), results show a better matching performance of the proprioceptive target.

**Table 4:** Statistical *p*-values for the matching error between the seven perturbations.

		*******JPM*******_*********UP*********_		*******JPM*******_*********PP*********_	
**PS [**^****°****^**]**		**P (AA)**	**P (FE)**	**P (AA)**	**P (FE)**
**45**					
	20	0.377	0.473	0.145	0.114
	5	0.296	0.813	0.023*	0.152
	0	0.351	0.987	0.008*	0.860
	−45	0.409	0.323	0.478	0.810
	−20	0.312	0.863	0.061	0.160
	−5	0.318	0.522	0.019*	0.246
**20**					
	5	0.666	0.198	0.020*	0.963
	0	0.724	0.300	0.002*	0.120
	−45	0.828	0.770	0.766	0.099
	−20	0.526	0.513	0.456	0.711
	−5	0.527	0.946	0.027	0.741
**5**					
	0	0.754	0.802	0.854	0.09
	−45	0.458	0.245	0.031	0.058
	−20	0.866	0.635	0.182	0.975
	−5	0.926	0.194	0.947	0.793
**0**					
	−45	0.483	0.577	0.028	0.805
	−20	0.620	0.977	0.067	0.104
	−5	0.613	0.332	0.804	0.083
**−45**					
	−20	0.328	0.245	0.200	0.074
	−5	0.350	0.692	0.069	0.131
**−20**					
	−5	0.883	0.483	0.157	0.861

* *represents significant differences between the two perturbations compared*.

## Discussion

Understanding how proprioceptive information is encoded at distal joints, has multiple intersections across different fields involving physiology, motor learning, sensorimotor recovery as well as those applications in haptics where proprioception is predominantly involved in a robot mediated manipulation. In rehabilitation practice, it is a common opinion among clinicians that current proprioceptive assessment fails in providing a reliable and quantitative information which would allow to compare motor and sensory deficits, known to be complementary information to a comprehensive diagnosis of the recovery process. However, authors usually focus on motor recovery (Soekadar et al., 2019) while limited evidence can be found in literature on the physiology of proprioception involving distal joints at the level of hand and wrist, despite they are anatomical districts covering an essential role in manual handling, and being the joints mostly involved in fine manipulation and exploitation of human dexterity, which is still unmatched in nature among species (Hoseini et al., 2015; Moser et al., 2020). With this in mind, we wanted to provide further evidences that using haptics, proprioceptive acuity can be accurately and geometrically characterized across the wrist's DoFs, synergistically involved during motor coordinated activities.

Hence, we decided to investigate if perturbations along one wrist joint (PS), can significantly alter the mechanism underlying perception of proprioceptive information on the adjacent DoFs (FE and AA). Outcomes revealed multiple aspects, which, to our knowledge have never been reported in previously published contributions, for the reason that most of the literature on proprioception primarily focused on proximal joints—shoulder and elbow—and privileged research on influence and role of multisensory integration in goal directed movements. Another reason for such lack of results, is the affordability of complex haptic devices, which not only assume operators able to skillfully program and run specific tailored physiological tests, but also they must be designed in such a way to provide robust and accurate position/force rendering and at the same time perform as reliable measurement systems.

By introducing a different order of presentation of the proprioceptive targets and disturbance input, we tried to understand if proprioceptive information is stored by the central nervous system in an absolute or relative coordinates frame. In our hypothesis the rotation of the reference system during or after the presentation of a target could have affected the final performance. Results clearly highlighted that mechanisms underlying the encoding of a proprioceptive target does not depend on the temporal order of the superimposed geometrical conditions; subjects are, in fact, able to store sequence of joints' configurations and to replicate, with the same accuracy, a previously experienced proprioceptive target independently on the initial conditions in which the target is presented and encoded.

We also found that proprioceptive acuity varies across DoFs: previously published works (Cappello et al., 2015; Marini et al., 2016a) experimentally demonstrated the existence of wrist proprioceptive anisotropy among its DoFs. Marini et al. (2016a) provided a map of the wrist position sense across each DoF, by means of the same robotic device used in our study, observing that wrist AA has a higher proprioceptive acuity respect to the remaining DoFs. Our results are in accordance, but also provide a wider perspective, reporting evidences that proprioception at the distal and multi-joint level, might be highly influenced by the mutual configuration between the DoFs composing the wrist anatomical joint, when the provided proprioceptive targets differ in amplitude across each DoF.

In details, the quantification of wrist anisotropy across its workspace and the dependence on initial posture, demonstrate that our peripheral sensory system tunes its sensitivity depending on geometric conditions and independently from the order of their presentation. Results clearly show a higher proprioceptive acuity for large perturbation amplitude, when the pronation supination (PS) was rotated ∓45°. We found the lowest value of the *Matching Error* for both AA and FE when the maximum wrist PS perturbation of ∓45° was applied, unexpectedly meaning that the neutral physiological posture of the forearm (zero rotation of the PS) is not a configuration which enables the best proprioceptive sensitivity. This effect finds its explanation when considering the mutual relationship between the activation of the mechanoreceptors, the anatomical structures of the muscular and connective tissues that are instrumental in proprioceptive coding (van der Wal, 2009). The aforementioned parts cannot be divided into either joint receptors or muscle receptors when muscular and connective tissues work in series to maintain joint integrity and stability: this happens at the boundary of their workspace.

It is known that joint receptors are highly reactive at the extremes of joint workspace (Ferrell et al., 1987), when the joint capsule is significantly stressed (McCloskey, 1978), for example (in our experiment) when the wrist is rotated at ∓45° along PS axis. The activation of the joint receptors, induced by the connective tissues after the changes in muscle tension, occurs at the limits of wrist' range of motion (van der Wal, 2009), and it might be responsible for the high proprioceptive acuity.

In our study there are anyway limitations: the first concerns the small sample of subjects included in our experimental sessions. Another limitation mostly refers to the number of trials provided for each DoF, which has been limited in order to avoid longer sessions with consequent loss of attention from the subjects. In order to deeply correlate joint- and mechano-receptor activation, proprioceptive acuity and perturbations, other measurements, such as surface electromyography (Mugnosso et al., 2018), could have been included in order to highlight the physiological aspects in terms of bio signals and not merely relying on kinematic data extracted by the haptic device. At last, since the current study investigates the influence of static wrist posture variation on proprioceptive acuity, future research could explore how sensory information is coded when time-variable dynamic conditions are provided.

We also mentioned in the introduction the possible application of the proposed paradigm for clinical settings: we believe that using a neuroergonomic haptic technology for quantification of sensory impairment is a viable option. Our approach was meant to analyze the proprioceptive anisotropy across the different DoFs of the wrist workspace, in particular for healthy subjects. Yet the methodological approach must be tailored in such a way to design a more compact test which can be dispensed on patients where physiological conditions are unpredictably variable and heterogeneous.

## Conclusion

This study aims at providing a wider and more comprehensive view on the physiological aspects influencing proprioception in the complex multi-joint articulation of the human wrist by means of a neuroergonomic robotic technology.

The outcomes are of interest for multiple disciplines: in neuroergonomics and medicine, for instance, the tests assessing sensory system's integrity, must be performed considering that different postural conditions may alter proprioceptive acuity. Testing patients' proprioception in a configuration which is close to the joints' physiological workspace limits, may increase mechanoreceptors excitation and provide a fine measurement of sensory acuity.

In haptics, especially for those applications where telemanipulation of real or virtual objects are mediated by robotic devices (robot aided surgical intervention), small movement of the master can be better perceived and controlled by the operator if her/his proprioception is set to a high sensitivity level and therefore in a posture with is proximal to the physiological boundaries of the joints' workspace.

## Data Availability Statement

The datasets generated and/or analyzed for this study are available from the corresponding author on reasonable request.

## Ethics Statement

The studies involving human participants were reviewed and approved by Heidelberg University Institutional Review Board (S-287/2020). The patients/participants provided their written informed consent to participate in this study. Written informed consent was obtained from the individual(s) for the publication of any potentially identifiable images or data included in this article.

## Author Contributions

ED'A and EG helped to conceive the idea and concept, they designed and implemented the experiment, acquired the data, analyzed and interpreted the data, and drafted the manuscript. JZ technically supported the development of the robotic application. FP, JK, and MC critically revised the manuscript content. LM conceived the idea and concept, critically revised the manuscript content, and supervised the study. All authors read and approved the final manuscript.

## Conflict of Interest

The authors declare that the research was conducted in the absence of any commercial or financial relationships that could be construed as a potential conflict of interest.
